# Clinical perspectives in congenital adrenal hyperplasia due to 3β-hydroxysteroid dehydrogenase type 2 deficiency

**DOI:** 10.1007/s12020-018-01835-3

**Published:** 2019-02-04

**Authors:** Abdullah M. Al Alawi, Anna Nordenström, Henrik Falhammar

**Affiliations:** 1 Department of Medicine, Sultan Qaboos University Hospital, Muscat, Oman; 2 Division of Medicine, Royal Darwin Hospital, Darwin, NT Australia; 3 Department of Women’s and Children’s Health, Karolinska Institutet, Stockholm, Sweden; 4 Department of Paediatric Endocrinology, Astrid Lindgren Children Hospital, Karolinska University Hospital, Stockholm, Sweden; 5 Department of Endocrinology, Metabolism and Diabetes, Karolinska University Hospital, Stockholm, Sweden; 6 Department of Molecular Medicine and Surgery, Karolinska Institutet, Stockholm, Sweden; 7 Menzies School of Health Research, Darwin, NT Australia

**Keywords:** 3βHSD2D, Diagnosis, Management, Outcomes, Mutations, Dehydroepiandrosterone

## Abstract

**Purpose:**

3β-hydroxysteroid dehydrogenase type 2 deficiency (3βHSD2D) is a very rare variant of congenital adrenal hyperplasia (CAH) causing less than 0.5% of all CAH. The aim was to review the literature.

**Methods:**

PubMed was searched for relevant articles.

**Results:**

3βHSD2D is caused by *HSD3B2* gene mutations and characterized by impaired steroid synthesis in the gonads and the adrenal glands and subsequent increased dehydroepiandrosterone (DHEA) concentrations. The main hormonal changes observed in patients with 3βHSD2D are elevated ratios of the Δ5-steroids over Δ4-steroids but molecular genetic testing is recommended to confirm the diagnosis. Several deleterious mutations in the *HSD3B2* gene have been associated with salt-wasting (SW) crisis in the neonatal period, while missense mutations have been associated with a non-SW phenotype. Boys may have ambiguous genitalia, whereas girls present with mild or no virilization at birth. The existence of non-classic 3βHSD2D is controversial. In an acute SW crisis, the treatment includes prompt rehydration, correction of hypoglycemia, and parenteral hydrocortisone. Similar to other forms of CAH, glucocorticoid and mineralocorticoid replacement is needed for long-term management. In addition, sex hormone replacement therapy may be required if normal progress through puberty is failing. Little is known regarding possible negative long-term consequences of 3βHSD2D and its treatments, e.g., fertility, final height, osteoporosis and fractures, adrenal and testicular tumor risk, and mortality.

**Conclusion:**

Knowledge is mainly based on case reports but many long-term outcomes could be presumed to be similar to other types of CAH, mainly 21-hydroxylase deficiency, although in 3βHSD2D it seems to be more difficult to suppress the androgens.

## Introduction

Congenital adrenal hyperplasia (CAH) is a group of disorders caused by deficiency of one of five enzymes that are responsible for making cortisol from cholesterol in the adrenal glands [1–3]. 21-hydroxylase deficiency (21OHD) is the most common disorder causing CAH (95-99%) followed by 11-beta-hydroxylase deficiency (11OHD) [2, 4–7].

3β-hydroxysteroid dehydrogenase type 2 deficiency (3βHSD2D) [8, 9], is a very rare type of CAH affecting <0.5% of all CAH [4, 5], and with <1/1,000,000 estimated prevalence at birth [10]. This disorder is caused by *HSD3B2* gene mutations and characterized by impairment of steroid synthesis in the gonads and the adrenal glands [11]. This leads to decreased cortisol, aldosterone, and androstenedione concentrations, however, renin, ACTH, and dehydroepiandrosterone (DHEA) concentrations are increased with DHEA being converted to testosterone by extra-adrenal 3βHSD1 [2]. The first cases of 3βHSD2D were reported by Bongiovanni in USA 1962 [12]. The clinical presentation varies according to the type (severity) of the genetic mutation and may include salt-wasting (SW) in both sexes, incomplete masculinization in males, and virilization in females. Elevated Δ5-17-hydroxypregnenolone is the best single biological marker or indicator of 3βHSD2D [13], but molecular genetic testing is recommended to confirm the diagnosis [14]. Glucorticoid and mineralocorticoid replacement therapy constitutes the main treatment [15]. In addition, sex hormones may be required for some patients who fail to progress through puberty [16].

The aim of this review is to provide a summary of currently available knowledge of CAH due to 3βHSD2D.

## Physiology

**Fig. 1:**
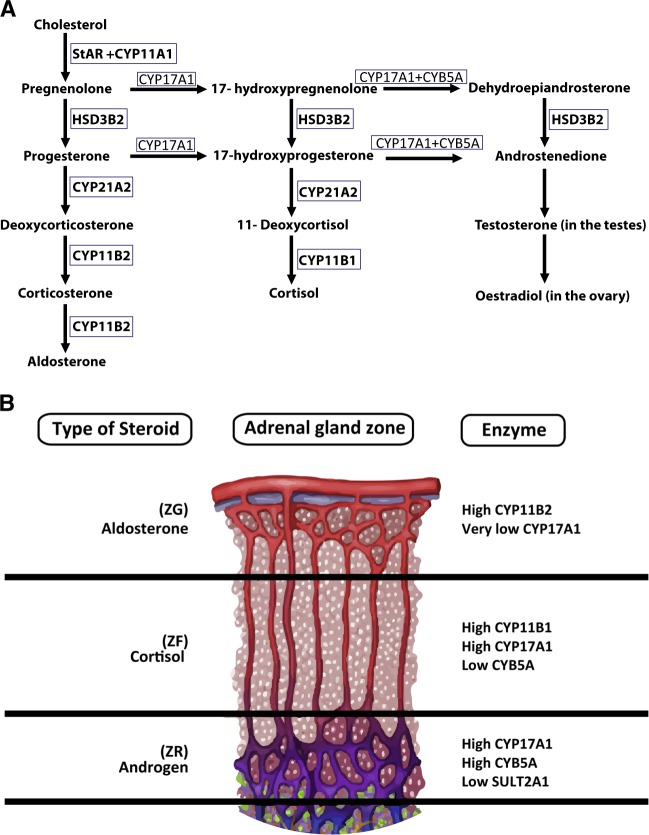
**a** Normal steroidogenesis in the adrenal cortex. The pathways of aldosterone, cortisol, and androgen synthesis and the enzymatic steps from the precursor cholesterol are shown. **b** Adrenal hormonal synthesis and enzyme expression pattern. ZG zona glomerulosa, ZF zona fasciculata, ZR zona reticularis, CYP11B2 aldosterone synthase, CYP17A1 17α-hydroxylase/17,20-lyase, CYP11B1 11β-hydroxylase, CYB5A, cytochrome b5, SULTA1 steroid sulfotransferase type 2A1

The adrenal glands are vital organs where steroidogenesis (in adrenal cortex) and catecholamine production (adrenal medulla) take place. The adrenal cortex has three compartments: zona glomerulosa, zona fasciculata, and zona reticularis [17] (Fig. 1). In the first step of the steroidogenesis StAR transports the cholesterol across the membrane, and then cholesterol is converted pregnenolone by the P450 side chain cleavage enzyme [18]. Within the zona glomerulosa, HSD3B2 converts pregnenolone to progesterone, which eventually is converted into aldosterone by a series of enzymatic processes involving CYP21A2 and aldosterone synthase [17]. In the zona fasciculata, CYP17A1 hydroxylates pregnenolone to form 17-hydroxyprogesterone (17OHP) which is then converted via several enzymes, including CYP11B1 and HSD3B2, to form cortisol. In the zona reticularis, 17-hydroxypregnenolone is converted to DHEA by CYP17A1. Then, HSD3B2 converts DHEA to androstenedione, which is a precursor of sex hormones [18]. The conversion of the Δ5-3β-hydroxysteroids (pregnenolone, 17-hydroxypregnenolone, and DHEA) to a Δ4-3-ketosteroids (progesterone, 17OHP, and androstenedione) by HSD3B2 involves dehydrogenation followed by an isomerization reaction [19]. Similarly, within the Leydig cells in the testis, cholesterol is converted to pregnenolone, 17-hydroxypregnenolone, DHEA and androstenedione. HSD17B3/AKR1C3 converts androstenedione or androstenediol to testosterone [20]. DHEA is converted by SULT2A1 to the more stable sulfated form (DHEAS). DHEAS has longer half-life (<10 h) and only 20% diurnal variation (DHEA ~30 min and 300%, respectively) [21] and is therefore measured more often than DHEA, for practical reasons. DHEAS can then be reconverted to DHEA by steroid sulfatase (STS) (~70%) but also to a certain degree by SULT2A1 located in the liver and kidney [22, 23].

### Pathophysiology

**Fig. 2:**
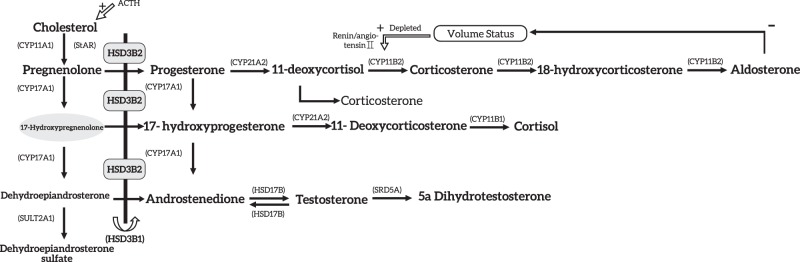
Pathophysiology in 3β-hydroxysteroid dehydrogenase type 2 deficiency

21OHD and 11OHD impair steroidogenesis in the adrenal glands only [1–3, 6, 17]. In contrast, severe form of 3βHSD2D causes defects of steroidogenesis in both adrenal glands and gonads [2, 3, 15]. Figure 2 illustrates the pathophysiology of 3βHSD2D.

HSD3B2 catalyzes reactions responsible for synthesis of a 3-keto-Δ4 A-ring, which is an essential part of endogenous mineralocorticoids, glucocorticoids, progestins, and androgens [3, 12, 24]. As a result, 3βHSD2D impairs the synthesis of progesterone, the precursor hormone of aldosterone, 17OHP, the precursor for cortisol, androstenedione, testosterone, and estrogen in the adrenal glands and gonads [13, 24]. Reduced levels of cortisol decrease the negative feedback on the pituitary gland causing excess ACTH production. Subsequently, ACTH drives the accumulation of β-hydroxy-Δ5-steroids pregnenolone, 17-hydroxypregnenolone, and DHEA, and their sulfates [25]. These precursor steroids cannot compensate for the cortisol and aldosterone deficiencies resulting in electrolyte disturbances and SW in most patients [12]. In the peripheral tissues, the intact isoenzyme HSD3B1 enzyme converts circulating DHEA to testosterone [16].

Elevated level of androstenedione leads to relatively high level of testosterone in females, however, it fails to achieve full compensation for absence of testosterone synthesis in males. In 46,XY neonates testosterone deficiency causes genital ambiguity. On the other hand, in 46,XX neonates, the relatively high level of testosterone may cause clitoromegaly and partial labioscrotal fusion. In addition, undiagnosed females may present with precocious pubarche, acne, hirsutism, and menstrual disturbances [26].

The human type I isoenzyme 3βHSD is the isoenzyme, encoded by *HSD3B1* gene and is expressed in peripheral tissue including skin, mammary glands, and placenta [9, 11, 14]. It has 372 amino acids and shares more than 90% homology with the type II isoenzyme [27, 28]. The human type I isoenzyme 3βHSD catalyzes transformation of DHEA into sex steroids including testosterone and estradiol [9].

### Clinical presentation

The phenotype of 3βHSD2D varies according to the genetic defect from severe SW form in neonates to mild menstrual disorders in older females [13, 15, 20].

### Incomplete masculinization in males

In normal 46,XY fetuses, androgens are required for penile development including the urethra and fusion of the labial-scrotal folds that normally takes place before 12 weeks of gestation [29]. Severe form of 3βHSD2D is associated with varying manifestations of incomplete masculinization including severe hypospadia, micropenis, bifid scrotum, and undescended testis [16, 20, 29].

### Female virilization

Depending on the genetic mutations, 46,XX infants can show enlarged clitoris, incomplete labial fusion and genital hyperpigmentation [30]. In contrast, some girls can have normal external genitalia which may delay diagnosis and they can subsequently present with adrenal crisis [31]. Older girls and women with genetically confirmed non-SW 3βHSD2D can present with androgen symptoms of hirsutism, premature pubarche or menstrual disorders including oligomenorrhea and primary amenorrhea [16, 32].

### Salt-wasting

Several deleterious mutations in the *HSD3B2* gene have been described that can cause SW during the first few weeks of life and may be fatal if not treated adequately [31, 32]. Biochemical findings include hyponatremia, hyperkalemia, metabolic acidosis and hypoglycemia [15, 33]. On the other hand, missense mutations in the coding region of *HSD3B2* gene is associated with non-SW form due to the presence of some residual enzymatic activity, about 10%, is sufficient to prevent aldosterone deficiency [16, 24, 32, 34, 35].

### Hypoglycemia

Recurrent episodes of hypoglycemia were reported to be a presenting feature in a suspected case of 3βHSD2D but the genotype was not performed to confirm the diagnosis [36]. Another patient presented during second day of life with hypoglycemia, later on, the molecular genetics confirmed 3βHSD2D [31].

### Diagnosis

In case of SW phenotype, 3βHSD2D is usually diagnosed within the first few weeks of life. In case of non-SW phenotype, patients may be diagnosed at any time before puberty [37]. However, the diagnosis has rarely been further delayed and patients can present with gender role related concerns during adulthood [38]. Overall, the patients tend to be diagnosed at a younger age in 46,XY children due to a higher rate of genital ambiguity compared to females [34, 39]. Also, there seems to be an underrepresentation of 46,XX patients, which might be explained by lack of diagnosis in milder form of 3βHSD2D in females. Also, females with severe form may die undiagnosed in a neonatal adrenal crisis more often than males [15].

When adrenal insufficiency is suspected in the setting of an adrenal crisis (i.e., an acute hemodynamic disturbance with hyponatremia, hyperkalemia and often hypoglycemia), blood should be drawn for steroid hormone measurements [15], but without delaying the lifesaving acute treatment with intravenous (or intramuscular) hydrocortisone [40, 41]. Low cortisol with high ACTH is consistent with primary adrenal insufficiency [42].

As 3βHSD2D catalyzes the conversion of Δ5-steroids (pregnenolone, 17-hydroxypregnenolone, DHEA, androstenediol) to Δ4-steroids (progesteron, 17OHP, androstenedione, testosterone), the main hormonal changes observed in patients with 3βHSD2D are high ratios of the Δ5- over Δ4-steroids [24, 43]. This includes raised 17-hydroxypregnenolone to 17OHP and DHEA(S) to androstenedione ratios in serum, and pregnanetriol to pregnanediol ratio in urine [15, 44, 45].

### ACTH stimulation test and hormonal profiles

Morning administration of 250 μg of synthetic ACTH followed by measurements of plasma Δ5-17-hydroxypregnenolone (5–17P), cortisol, Δ4-17-hydroxyprogesterone (17OHP), DHEA(S), and androstenedione can be used to improve the diagnostic process of 3βHSD2D [3, 13]. Hormonal criteria for the diagnosis of 3βHSD2D have been developed from a previous study [13], where hormonal profiles of patients with homozygous/compound heterogeneous *HSD3B2* mutations and people with normal *HSD3B2* genes were compared. ACTH stimulation test shows, apart from diminished cortisol, an exaggerated response and high level of Δ5-17-hydroxypregnenolone in patients with homozygous/compound heterozygous *HSD3B2* mutations and varies according to patient age (Table 1) [13, 46].

**Table 1:** Post ACTH stimulation test of Δ5-17-hydroxypregnenolone in patients with 3βHSD2D confirmed by *HSD3B2* mutation analysis [13]

Neonates with ambiguous genitalia	≥378 nmol/L
Tanner stage I children with ambiguous genitalia	≥165 nmol/L
Children with premature pubarche	≥294 nmol/L
Adults	≥289 nmol/L

In general, Δ5-17-hydroxypregnenolone above 100 nmol/L, either basal or after ACTH stimulation, is the best single biological criterion of 3βHSD2D [16, 31, 37]. The hormonal profile cannot distinguish heterozygous carriers from normal people [3, 47].

Other biochemical findings are elevated renin, relatively high level of testosterone in girls, elevated 17OHP (via peripheral conversion, see below), elevated DHEA(S), elevated urinary Δ5-OHP, and DHA metabolites [16].

### Molecular analysis and genetic studies

There are two isoenzymes of human 3βHSD which are encoded by different genes located on the p13.1 region of chromosome 1 [14, 15]. Both genes are included within a DNA fragment of around 7.8 kB and consist of four exons and three introns [19, 24, 34]. The *HSD3B2* gene encodes the human type II 3βHSD isoenzyme and is expressed in the adrenal cortex and in the gonads. The isoenzyme is essential for the adrenal synthesis of glucocorticoids, mineralocorticoids, and sex steroids [9, 10, 34, 36]. More than 40 mutations have been found in the *HSD3B2* gene causing 3βHSD2D and a few of them have been identified in isolated populations (Table 2) [10, 15, 16, 20, 24, 29–31, 35, 37, 48–64].

**Table 2:** *HSD3B2* gene mutations causing 3β–hydroxysteroid dehydrogenase type 2 deficiency

Mutation/genotype	Sex	Clinical presentation	Comments	First author [reference]
Homozygous mutation c.73G >T(p.E25X)	Female	SW Mild virilization		Huang [61]
(L205P, p.Leu205Pro)	Male	SW Hyperpigmentation Severe hypospadias Bifid scrotum	No detectable 3βHSD activity	Moisan [16]
Compound heterogeneous mutation 186/insC/187 and (Y253N, p.Tyr253Asn)	Male	SW Severe undervirilization Hypospadia	Frames shift, missense No detectable 3βHSD activity	Simard [24, 29]
Compound heterogeneous mutation: W171X/(E142K, p.Glu142Lys)	Male	SW Severe undervirilization Hypospadia	Nonsense, missense No detectable 3βHSD activity	Simard [24, 29]
(A82P, p.Ala82Pro)	Male	SW Perineal hypospadias		Rabbani [60]
Homozygous mutation 687del27	Male	Neonatal SW Micropenis with a perineal hypospadias	Achieved normal puberty Adult spermatic characteristics were normal	Donadille [10]
687del27 homozygous mutation	Male	Perineal hypospadias Miropenis SW	No detectable 3βHSD activity	Moisan [16]
Homozygous c.687del27	Male	Severe undervirilization Low steroid production Arrested spermatogenesis Gynecomastia		Burckhardt [20]
Compound heterogeneous mutation 318 [ACA (Thr)] —>AA 273 [AAA(Lys) —>A]	Female	SW Sexual ambiguity		Zhang [30]
(T259M, p.Thr259Met)	Male	Perineal hypospadia Bifid scrotum SW	No detectable 3βHSD activity	Moisan [16]
	Female	Mild clitromegaly Premature pubarche		Marui [35]
(T259R, p.Thr259Arg)	Male	Pigmentation Hypospadias Bifid scrotum SW	No detectable 3βHSD activity	Moisan [16]
	Female	Normal genitalia with severe pigmentation SW		
Compound heterozygote A82D, W230X	Female	Hypoglycemia SW		Nordenstrom [31]
(P222Q, p.Pro222Gln)	Male	Perineal hypospadias Micropenis SW	No detectable 3βHSD activity	Moisan [16]
(P155L, p.Pro155Leu)	Male	Perineal hypospadias SW	No detectable 3βHSD activity	Moisan [16]
Homozygous p.W355R (c.763 T>C)	Male	Hypospadias cryptorchidism Bifid scrotum SW	TART	Guven [63]
(A10E, p.Ala10Glu)	Male	Sexual ambiguity SW Azoospermia	Missense	Alos [15]
	Female	Normal genitalia SW/normal puberty		
Homozygous p.Q334X (c.1000C>T)	Male	SW Hypospadias, small phallus, bifid scrotae, palpable gonads	TART	Alswailem [28]
	Female	SW Normal genitalia		
p.R335X (c.1003C>T)	Male	SW Hypospadias Bifid scrota Palpable gonads Advanced bone maturation	Bilateral TARTs	
	Female	SW Normal genitalia		
W171X :Trp171 Stop	Female	SW Normal external genitilia Failure of breast development	Nonsense	Rheaume [11]
Compound heterogenous mutation W171X: Trp171 Stop and 186/insC/187	Male	SW Hypospadias	Nonsense Adequate spermatogenesis	Rheaume [11]
273ΔAA	Male	SW Ambiguous genitalia	Frameshift mutation No residual enzymatic activity	Simard [48]
Compound heterogenous mutation (L108W, p.Leu108Trp) (P186L, p.Pro186Leu)	Male	SW Hypospadias	Missense Less than 0.5% enzymatic activity	Sanchez [53]
(G15D, p.Gly15Asp)	Male	SW Hypospadias	Missense	Rheaume [49]
Compound heterozygous for T181I1 and 1105delA	Female	SW Premature pubarche, slight growth acceleration, and advanced bone age	Frameshift	Johannsen [37]
(P222T, p.Pro222Thr)	Female	SW	Missense	Pang [58]
(P341L, p.Pro341Leu)	Male	SW Micropenis		Welzel [59]
Heterozygosity.244G>A (p.Ala82Thr), 931C>T(p.Gln311*)	Female	Ambiguous genitalia		Teasdale [64]
(S213G, p.Ser213Gly)	Female	Premature pubarche at 4 y Growth acceleration	Detectable activity	Moisan [16]
(A245P, p.Ala245Pro)	Male	Sexual ambiguity	Detectable activity	Simard [24, 29]
(A10V, p.Ala10Val)	Male	Perinoscrotal hypospadia	Detectable activity (30%)	Moisan [16]
(L236S, p.Leu236Ser)	Male	Perinoscrota hypospadias Micropenis	Missense	Moisan [16]
	Female	Premature pubarche	Missense	Nayak [57]
(A245):Ala245Pro	Male	Hypospadias Bifid scrotum	Missense Detectable enzymatic activity	Simard [24]
(G129R, p.Gly129Arg)	Male	Perineal hypospadias	Missense/splice Detectable enzymatic activity	Rheaume [51]
	Female	Normal genitalia Premature pubarche		
(N100S, p.Asn100Ser)	Male	Perineal hypospadias	Missense	Mebarki [55]
(Y254D, p.Tyr254Asp)	Female	Severe acne Primary amenorrhea Clitoromegaly Moderate hirsutism	Missense	Sanchez [54]
(L173R, p.Leu173Arg)	Male	Perineal hypospadias	Missense Raised as female	Russel [52]
(A82T, p.Ala82Thr)	Female	Some with no signs of CAH One patient with premature pubarche	Missense	Mendonca [50]
	Male	Perineal hypospadias		
p.G250V	Female	Precocious pubarche Postnatal clitoromegaly		Baquedano [62]
(A167V, p.Ala167Val)	Female	Premature pubarche	Missense	Moisan [16]
(K216E, p.Lys216Glu)	Female	Premature pubarche	Missense	Moisan [16]
(P22H, p.Pro221His)	Female	Premature pubarche		Moisan [16]
(G294V, p.Gly294Val)	Male	Hypospadias		Moisan [16]

*SW* Salt wasting

In general, frameshift mutations, in-frame deletions, and nonsense mutations introducing a premature termination codon are associated with severe form of 3βHSD2D resulting in SW phenotype [14, 34, 65]. The locations of these mutations suggest that at least the first 318 amino acids out of 371 are required for HSD3B2 activity [14]. In contrast, missense mutations are associated with some residual enzymatic activity and non-SW phenotype [14]. There have been no reported mutations of the *HSD3B1* gene in human so far [32, 44].

### Neonatal screening

Newborns with atypical external genitalia should undergo hormonal profile analysis prior to hospital discharge to avoid presentation with SW crisis [66, 67]. Neonatal screening for 21OHD by detecting elevated level of 17OHP has been implemented in most developed countries [68]. 3βHSD2D can result in an increase in the level of circulating 17OHP due to peripheral conversion of high levels of accumulated Δ5-steroids by the isoenzyme 3βHSD type 1. There have been previous case reports of false positive, for 21OHD, neonatal screen for infants with 3βHSD2D [31, 65]. Accordingly, neonates with elevated 17OHP should undergo molecular genetic confirmation to confirm the type of enzymatic deficiency [14, 31, 68].

### Non-classic form of 3βHSD2D

Prior to the implementation of molecular genetic studies, it was thought that many children with premature pubarche, and females with hirsutism and menstrual irregularities might have a mild, late-onset (non-classic) form of 3βHSD2D [35, 37, 45]. This was supported by controversial hormonal criteria based on exaggerated Δ5-steroid production after ACTH stimulation test and elevated 17OHP to cortisol ratios [32]. However, genetic studies failed to detect any mutations in the *HSD3B2* gene in this group of patients [24, 29, 35, 37], and treatment with glucocorticoids and mineralocorticoids did not improve signs of androgen excess [29, 32]. A previous report has shown normalization of the hormonal profile after treatment with GnRH agonist for two patients diagnosed with polycystic ovarian syndrome (PCOS) associated with 3βHSD2D [69]. The exact mechanism of exaggerated Δ5-steroid production after ACTH stimulation is not clear and it might be related to a form of PCOS or other unidentified mechanism causing alteration in intra-adrenal 3βHSD activity [32]. A not uncommon presentation among adult women with mild hyperandrogenism is that they are found to have elevated serum DHEAS and/or reported to have “partial 3βHSD2D”, based on urine steroid profiling but with no *HSD3B2* gene mutations identified. The diagnosis usually ends up being PCOS. Thus, non-classic 3βHSD2D, if it exists, is extremely rare [2], in contrast to non-classic 21OHD [70, 71].

### Pubertal status

Few patients have been evaluated after puberty [15, 20, 33, 72–75]. With good compliance with glucocortiocid and mineralocorticoid replacement therapy [15], most 46,XX patients have shown progressive feminization at appropriate age with menstruations [15, 33, 74]. In contrast, one female with severe *HSD3B2* mutations had minimal breast development at age 14.7 years, required gonadotropin injections and estrogen treatment to develop full feminization. However, with cessation of estrogen and progesterone replacement treatment, her menstrual cycle ceased and she developed ovarian cysts [16, 76].

The pubertal development has been reported in some males with *HSD3B2* mutations. Most of these patients entered puberty spontaneously without need for testosterone supplementation [15, 20, 33, 72, 74, 75, 77]. This could be explained by peripheral conversion of DHEAS to testosterone by HSD17B5 activity [10, 20].

### Gynecomastia

In adult males with 3βHSD2D, HSD3B1 converts the high amount of androgen precursors (DHEA and androstenediol) in peripheral tissues to androstenedione or testosterone [20]. Then HSD17B1, HSD17B5, and CYP19A1 enzymes catalyze the conversion of androstenedione and testosterone to estrogens [20]. High level of estrogens is associated with gynecomastia in males [10, 20, 72]. Testosterone replacement therapy was found to reduce gynecomastia by suppressing gonadotrophin synthesis via negative feedback [20].

### Final height

Final height has been reported in a few patients and the adult height seemed to be within the target range when control of the hyperandrogenism during the growth period had been good [15], but otherwise the final height was reduced [75].

### Fertility

3βHSD is required for biosynthesis of not only mineralocorticoids and glucocorticoids, but also sex hormones. Accordingly, males with 3βHSD2D may suffer from decreased spermatogenesis and infertility. Also, females may have menstrual irregularity and infertility [20]. However, there is very limited information about fertility, semen analysis and testicular histology in patients with 3βHSD2D [15, 20, 73, 75]. In case reports of 46,XY patients, semen analyses have shown azoospermia [15, 75]. Moreover, testicular histology in adult males with 3βHSD2D showed spermatogenic arrest at the level of spermatogonia [20, 78]. In contrast, a patient with severe *HSD3B2* mutations, with annual follow-ups from birth until the age of 23 years old, demonstrated normal sperm production probably attributed to his good compliance with treatment [10]. This might suggest that fertility is possible even with severe mutations. One case of an adult male fathering two children has been reported, however, there was no genetic testing to confirm his paternity [10]. In 21OHD, fertility has been shown to be impaired in both females and males [4, 79–86], however, the fertility may be normal if the male has been diagnosed and treated early on since the neonatal period. If this is also true in 3βHSD2D is unknown.

### Testicular adrenal rest tumors

During abdominal surgery, the presence of ectopic adrenocortical tissue is a common incidental finding in otherwise healthy individuals without clinical significance [87]. In patients with CAH and during period of suboptimal treatment, high levels of ACTH and angiotensin II can stimulate adrenal-like cells causing development of testicular adrenal rest tumors (TARTs) and rarely ovarian adrenal rest tumors [75, 87]. The prevalence of TARTs varies between 34 and 94% according to different reports in males with CAH due to 21OHD [82, 85, 88, 89]. TARTs have been reported in some patients with 3βHSD2D [15, 63, 75], but it is difficult to estimate the prevalence. Also, it has been demonstrated that presence of TARTs has a negative impact on fertility in males with 21OHD [82, 88, 90]. Similarly, in previous case reports, males with 3βHSD2D and TARTs have been found to have severely impaired spermatogenesis [63, 75, 82]. High dose of corticosteroids might reduce the size of TART [63]. It has been recommended that all patients with CAH should undergo regular testicular examination with ultrasonography [1, 7, 90]. Even though these recommendations were primarily written for 21OHD it can be assumed that males with 3βHSD2D have equal benefits.

### Bone mineral density and fractures

Supraphysiological glucocorticoid replacement has harmful effects on bone mineral density (BMD) via multiple mechanisms [91, 92]. Only one case of 3βHSD2D and BMD measurements has been reported, and has showed osteoporosis [75]. In general, studies in adults with CAH have demonstrated impaired BMD [4, 93–100], even though there are exceptions with normal BMD [101, 102], and better than other DSD conditions [103]. Prednisolone may be associated with worse BMD than hydrocortisone [95, 97, 104, 105]. Fractures have not been reported in 3βHSD2D so far but may be increased in CAH in general [93, 95, 97, 99, 100, 103].

### Obesity, diabetes, and cardiovascular disease

Obesity, including severe, has been reported in patients with 3βHSD2D [63, 75], probably due to iatrogenic Cushing syndrome. It could be assumed that the prevalence of obesity, diabetes and cardiovascular disease in 3βHSD2D is similar to most other forms of CAH, most commonly 21OHD, and mainly due to glucocorticoid excess but androgen excess and/or deficiency may also contribute. The majority of studies including adults and children with CAH have reported an increased body fat mass assessed by DXA [96, 101, 102, 106, 107], which enables separation between lean mass (which may be increased due to hyperandrogenism) from fat mass. Elevated cardiometabolic risk, including insulin resistance [4, 94, 108–117], has been reported in a large number of studies on CAH, with a few reporting increased rate of established cardiovascular disease [103, 118], and diabetes (including gestational diabetes) [81, 109, 118]. Very few individuals with CAH above 50 years of age have been included in studies, and thus it could be expected that the rate will increase since cardiovascular disease and diabetes usually develop later in life [1].

### Psychiatric diseases

Psychiatric disorders have so far not been reported in studies with exclusively 3βHSD2D recruited [119]. In studies of CAH psychiatric diseases have only occasionally been investigated and these have shown an increased rate [103, 120, 121], especially of depression [122], alcohol misuse [120, 121], and suicidality [103, 120].

### Adrenal tumors

Chronic elevation of ACTH will lead to hyperplasia of the adrenal cortex and sometimes subsequent tumor formation [123–125]. Adrenal tumors have so far not been reported in 3βHSD2D but are known to affect 11–82% of patients with other CAH variants [124, 126, 127]. Adrenal incidentalomas, i.e., adrenal tumors found serendipitously by imaging for other reasons than suspected adrenal tumor or disease [128], have sometimes been the initial presentation of CAH, including classic CAH, both in case reports and adrenal incidentaloma cohorts [125, 129–134].

### Mortality

Very little is known about the mortality in individuals with 3βHSD2D. The introduction of glucocorticoid replacement and increased awareness have increased the survival of classic 21OHD [5], and this is most probably also the case in 3βHSD2D. In population studies, patients with CAH had generally an increased mortality rate (hazard ratio 3–5) and died 6.5–18 years earlier, compared to controls [122, 135]. Adrenal crisis was the main cause of death [135], iterating the importance of stress dosing during acute illness [40, 41]. Mortality studies in pure 3βHSD2D will probably never be performed due to its rareness.

### Treatment

Glucocorticoid and mineralocorticoid replacement is similar to other forms of CAH. In SW crisis, treatment includes prompt rehydration, correction of hypoglycemia, and parenteral hydrocortisone (intravenous or intramuscular) [15, 40, 41]. For follow-up children are treated with hydrocortisone in a dose of 10–15 mg/m^2^/day. Long-acting glucocorticoids such as dexamethasone and prednisolone, known to suppress growth in children, can be used during adulthood [7, 33, 67]. Compared to 21OHD it seems to be more difficult to suppress the androgens in 3βHSD2D, which could be speculated be due to the DHEAS as a constant source of DHEA, testosterone and DHT. This may result in a need for slightly higher doses of glucocorticoids in 3βHSD2D with subsequently more long-term negative outcomes. Mineralocorticoid replacement can be achieved with fludrocortisone 0.1 mg/day [33] with regular monitoring of plasma renin activity [1, 7, 67]. Sex hormone replacement therapy should be considered for patients who show delayed progression through puberty [16]. In addition, testosterone replacement therapy might be considered for male patients with testosterone responsive microphallus to augment penile growth [33]. Hormonal replacement therapy should be combined with regular clinical and biochemical evaluation of these patients [15]. Surgical intervention might be indicated in some circumstances including undescended testis [63], hypospadias repair [20], and severe genital virilization [136–138]. Bilateral adrenalectomy has occasionally been used in selective cases with 21OHD or 11OHD to better control hyperandrogenism and/or to be able to lower the glucocorticoid doses with similar control of the hyperandrogenism [139]. Its utility in 3βHSD2D is currently unclear.

## Conclusion

3βHSD2D is a very rare form of CAH and the phenotype varies according to the severity of the *HSD3B2* mutations. In severe forms, the neonate can present with SW crisis but the diagnosis can be delayed in mild forms until adolescence. Hormonal criteria for the diagnosis of 3βHSD2D have been developed and it was proposed that Δ5-17-hydroxypregnenolone above 100 nmol/L, either basal or after ACTH stimulation, is the best single biological criterion of 3βHSD2D. However, molecular genetic testing is recommended to confirm the diagnosis. Glucocorticoid and mineralocorticoid replacement are the main treatments. Sex hormone replacement and surgical corrective procedures may be indicated in some patients. On the basis of case reports, 3βHSD2D may be associated with infertility, obesity, osteoporosis, TARTs, and reduced final height. However, very little is known about mortality, cardiovascular health, mental health, and adrenal tumor risk due to the rareness of 3βHSD2D but can be presumed to be elevated, and similar to 21OHD. Although in 3βHSD2D it seems to be more difficult to suppress the androgens, subsequently leading to slightly higher glucocorticoid doses. This may result in more long-term negative outcomes.
